# Substituent Effects on the Radical Scavenging Activity of Isoflavonoid

**DOI:** 10.3390/ijms20020397

**Published:** 2019-01-18

**Authors:** Yan-Zhen Zheng, Geng Deng, Rui Guo, Da-Fu Chen, Zhong-Min Fu

**Affiliations:** 1 College of Bee Science, Fujian Agriculture and Forestry University, Fuzhou 350002, China; yanzhenzheng@fafu.edu.cn (Y.-Z.Z.); rui_0508@163.com (R.G.); fzmfafu126@126.com (Z.-M.F.); 2 Key Laboratory of Bioorganic Phosphorous Chemistry and Chemical Biology (Ministry of Education), Department of Chemistry, Tsinghua University, Beijing 100084, China; dengg@mail.tsinghua.edu.cn

**Keywords:** isoflavonoid, substituent effect, structure-antioxidant activity relationship, Hammett sigma constants, density functional theory, antioxidant mechanism

## Abstract

Understanding the role of substituents is of great importance for the preparation of novel phenolic compounds with enhanced antioxidative properties. In this work, the antioxidative activity of isoflavonoid derivatives with different substituents placed at the C2 position was determined by density functional theory (DFT) calculations. The bond dissociation enthalpy (BDE), ionization potential (IP), and proton affinity (PA) related to hydrogen atom transfer (HAT), single electron transfer-proton transfer (SET-PT), and sequential proton loss electron transfer (SPLET) mechanisms were calculated. The strongest antioxidative group of isoflavonoid is not altered by the substituents. Excellent correlations were found between the BDE/IP/PA and Hammett sigma constants. Equations obtained from linear regression can be useful in the selection of suitable candidates for the synthesis of novel isoflavonoids derivatives with enhanced antioxidative properties. In the gas and benzene phases, the electron-donating substituents would enhance the antioxidative activity of isoflavonoids via weakening the BDE of 4′−OH. In water phase, they will reduce the antioxidative by strengthening the PA of 7−OH. Contrary results occur for the electron-withdrawing groups. In addition, the electronic effects of substituents on the BDE/IP/PA have also been analyzed.

## 1. Introduction

Oxidation reactions are the main reason for deterioration in biological systems and are related to ageing and diseases such as atherosclerosis, cancer, and Alzheimer’s disease [1]. This process arises from oxidative damage of biomolecules (such as proteins, nucleic acids, unsaturated lipids, and sugars) by the presence of reactive free radicals [2].

Antioxidants are chemical compounds that can trap reactive free radicals formed during oxidative reactions. In the past decades, interest in phenolic compounds has grown greatly, due to their free radical scavenging ability and other potential health benefits [3]. Isoflavonoids are a large class of phenolic compounds, mainly found in vegetables, fruits, cereals, and bee products [4,5]. Apart from their physiological performance in plants, isoflavonoids are also vital constituents in the human diet. In recent years, growing attention to isoflavonoids can be mainly ascribed to their free radical scavenging activities, which also greatly relates to other biological activities, such as antibacterial, antiviral, anti-inflammatory and anti-ischemic activities [5]. It is reported that isoflavonoids perform their anti-inflammatory actions mainly via the ability to modulate production of free radicals by phagocytic leukocytes [6].

It is well-established that phenolic compounds commonly scavenge free radicals via three possible pathways [7,8,9]. They are hydrogen atom transfer (HAT), single electron transfer-proton transfer (SET-PT), and sequential proton loss electron transfer (SPLET). From the antioxidative action viewpoint, the three mechanisms have the same net result: formation of phenoxy radicals and the termination of free radicals.

The antioxidative activity of phenolic compounds depends on their capacity to resist the detrimental effects of free radicals. It is related to the structure of the compound. Among multiple structural features, the substituent effect is one of the most important factors that influences the chemical, physicochemical, and biochemical properties of phenolic compounds [10,11,12,13]. Theoretical studies on the substituent effect can be utilized in the selection and synthesis of novel compounds with enhanced antioxidative activity. There are multiple studies in the literature that focus on the number and position of functional groups in relation to the biological activity of flavonoids [14,15,16]. On the other hand, the substituent effect on the antioxidative activity of isoflavonoids is seldom known.

Computational methods can provide valuable information about the biological performance of phenolic compounds. Due to their low cost, short time, and high precision performing calculations on the properties of phenolic compounds, density functional theory (DFT) calculations have been widely accepted as the most applicable computational methods for studying antioxidative activity. They have been successfully used to study the antioxidative activity and structure-activity relationships of phenolic compounds [7,8,9,17,18,19,20,21,22]. They can reveal the effects of different structural features on the antioxidative properties of phenolic compounds. The comparisons between the experimental and theoretical data are not only able to make the controversially experimental facts clear, but also able to design novel compounds with excellent antioxidative activity. In addition, DFT can also carry out the work that is difficult to perform by experiment.

**Figure 1:**
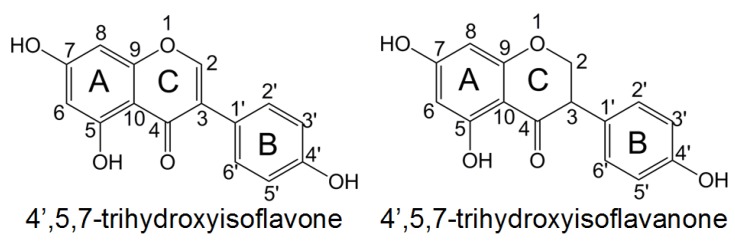
The chemical structures of 4′,5,7-trihydroxyisoflavone and 4′,5,7-trihydroxyisoflavanone.

Understanding the role of substituents is of great importance for the preparation of novel phenolic compounds with enhanced antioxidative properties. In this paper, the effects of various substituents on reaction enthalpies related to HAT, SET-PT, and SPLET mechanisms of isoflavonoid derivations were investigated by DFT method. The 4′,5,7-trihydroxyisoflavone and 4′,5,7-trihydroxyisoflavanone (Figure 1) are selected as the model molecule. Both of them have hydroxyl groups in rings **A**, **B**, and **C**, and thus can be used to study the substituent influence on the antioxidative activity of different rings. Various substituents (NH_2_, OMe, Me, OH, F, Cl, CHO, CF_3_, CN, and NO_2_) covering the electron-withdrawing groups and electron-donating groups were placed at the C2 position. To find a relationship between structural parameters and the antioxidative activity, correlations of calculated enthalpies with Hammett sigma constants of the substituents were investigated. Apart from the gas phase, benzene, and water phases were used as models to simulate environments (physiological lipids and fluids) found in many foods and body tissues.

## 2. Results

HAT is a one-step mechanism. SET-PT and SPLET mechanisms take place through two steps. Reaction enthalpies (bond dissociation enthalpy or BDE, ionization potential or IP, and proton affinity or PA) characterizing the first steps of three mechanisms are of great importance in evaluating the antioxidative action. Hence, the detailed analysis mainly focuses on the BDE, IP, and PA.

The Hammett sigma constant has been one of the most widely used parameters to study and interpret organic reactions and their mechanisms. In this work, the Hammett sigma constants *σ*_m_ and *σ*_p_ of the substituents were used. They were obtained from the ionization of organic acids in solutions and have been successfully used to predict the equilibrium and rate constants of a variety of families of reactions. The electronic effect of the substituent is mainly composed of two parts: the field/inductive effect (represented by parameter *F*) and the resonance effect (characterized by parameter *R*) [13,23,24]. In this work, the electronic effects of the substituents on the reaction enthalpies have also considered. Table 1 lists the *σ*_m_, *σ*_p_, *F,* and *R* of the studied substituents [13].

### 2.1. Bond Dissociation Enthalpy

In the HAT mechanism, the BDE is an important parameter evaluating the antioxidative action. It influences the effectiveness of the H-atom transfer reaction from the phenolic compounds to the reactive radical. The lower the BDE, the stronger the antioxidative activity and the more important the role of the corresponding O−H group in the antioxidative activity. The computed gas, benzene, and water phases of BDEs of 4−OH, 5−OH, and 7−OH for the studied compounds are listed in Table 2. As can be seen in Table 2, the lowest BDEs are at 4′−OH for the investigated compounds irrespective of the studied media. Therefore, the 4′−OH is the group most likely to undergo the HAT mechanism. In addition, the strongest antioxidative group of isoflavonoids does not alter by the substituents placed at C2, irrespective of the studied media.

**Figure 2:**
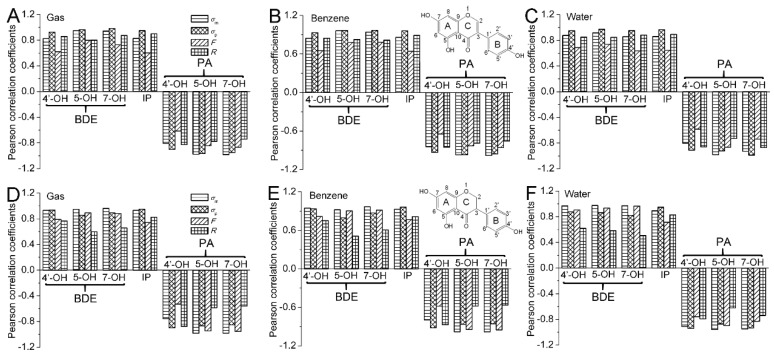
The Pearson correlation coefficients between the Hammett sigma constants (*σ*_m_ and *σ*_p_)/*F*/*R* and the BDE/ ionization potential (IP)/ proton affinity (PA) in the gas, benzene, and water phases.

The Pearson correlation coefficients between the *σ*_m_/*σ*_p_ and the BDEs are drawn as histograms in Figure 2. In Figure 2, all of the *P*(_BDE,*σ*_) are positive and larger than 0.8. Hence, the BDEs are positively and highly relevant with the Hammett sigma constants. The positive coefficient also characterizes how the electron-withdrawing groups in the C2 position raise the BDE, while electron-donating groups have the opposite effect. The obtained results can be interpreted to say that electron-withdrawing groups in the C2 position can stabilize the parent molecule and destabilize the radical. Hence, they increase the O−H BDE. However, electron-donating groups in the C2 position have the opposite effect, and their presence would lead to a decrease in the O−H BDE. Therefore, the electron-withdrawing groups placed at the C2 position would reduce the antioxidative activity of isoflavonoids, while the electron-donating groups have the opposite effect.

**Figure 3:**
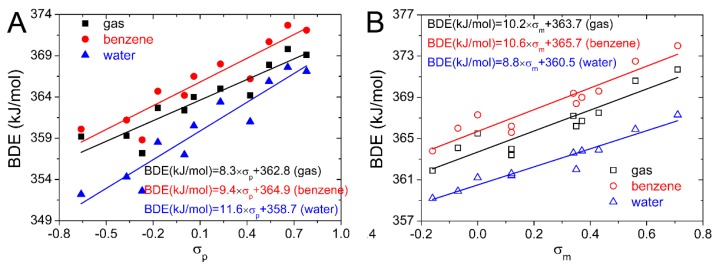
Dependence of BDE on Hammett sigma constants for 4′,5,7-trihydroxyisoflavone (**A**) and 4,5,7-trihydroxyisoflavanone (**B**) in gas, benzene, and water phases.

For O−H in 4′,5,7-trihydroxyisoflavone, the *P*(_BDE, *σ*p_) is larger than *P*(_BDE, *σ*m_) and exceeds 0.9. While for O−H in 4′,5,7-trihydroxyisoflavanone, the *P*(_BDE, *σ*m_) is larger than *P*(_BDE, *σ*p_) and are more than 0.9. Therefore, the correlation between the BDE in 4′,5,7-trihydroxyisoflavone and *σ*_p_ is better than that between the BDE and *σ*_m_ and the BDE of O−H in the substituted 4′,5,7-trihydroxyisoflavone can be predicted better by *σ*_p_. Meanwhile, an opposite result occurs in 4′,5,7-trihydroxyisoflavanone, and the BDE of O−H bond in substituted 4′,5,7-trihydroxyisoflavanone can be predicted better by *σ*_m_. In this work, BDE values of 4′−OH computed for the substituted isoflavonoids in the gas, benzene, and water phases are plotted against Hammett sigma constants in Figure 3. Equations obtained from the linear regression are also placed in Figure 3. They can be used to predict the BDEs of 4′−OH by the Hammett sigma constants. The hydroxyl group with the lowest BDE determines the antioxidative activity. As the 4′−OH is the strongest antioxidative hydroxyl group, thus the antioxidative activity of the substituted isoflavonoids can also be predicted by these equations.

The Pearson correlation between the *F*/*R* and the BDEs are also done and corresponding correlation coefficients are drawn as histograms in Figure 2. In Figure 2, it can also be observed that the correlation between the BDE of isoflavone and *R* is much better than that between the BDE of isoflavone and *F*. Meanwhile, a contrary result occurs for the BDE of isoflavanone. Therefore, the electronic effect of substituents at the C2 position on the BDE of 4′,5,7-trihydroxyisoflavone is mainly governed by the resonance effect, while that on the BDE of 4′,5,7-trihydroxyisoflavanone is mainly controlled by the field/inductive effect.

### 2.2. Ionization Potential

In a SET-PT mechanism, the first step is electron transfer. This step is significant for the SET-PT mechanism and it is characterized by IP. The lower the IP value, the higher the electron-donating ability, and the stronger the antioxidative activity. The computed gas, benzene, and water phase IPs for the basic structure and substituted isoflavonoids are listed in Table 3. Solvent causes considerable changes in the enthalpies of the ionic species. Thus, IP in the solvent phase is significantly lower than that in the gas phase, because of the fact that the cation radical is more stable and the conjugation of the π-electrons is more delocalized in the solvent media.

The Pearson correlation was carried out for the IP and Hammett sigma constants. As can be seen in Figure 2, the *P*(_IP,*σ*_) is positive and larger than 0.8 in the studied phases, indicating that the IP is positively and highly relevant with the Hammett sigma constant. The positive coefficient also suggests that electron-withdrawing groups raise the IP and electron-donating groups have an opposite effect. As is known in organic chemistry, the electron-withdrawing substituents can stabilize the parent molecule and destabilize the radical and cation radical. This results in an increase in IP. However, the electron-donating substituents have an opposite effect. Their presence in the molecule leads to a decrease in IP. Therefore, the electron-donating groups at the C2 position would enhance the antioxidative activity of isoflavonoids. At the same time, electron-withdrawing groups at the C2 position would play the opposite role in a SET-PT mechanism.

**Figure 4:**
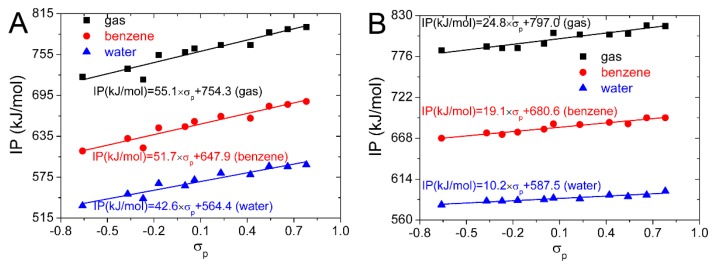
Dependence of IP on Hammett sigma constants for 4′,5,7-trihydroxyisoflavone (**A**) and 4′,5,7-trihydroxyisoflavanone (**B**) in gas, benzene, and water phases.

In Figure 2, the *P*(_IP, *σ*p_) is larger than *P*(_IP, *σ*m_), and is approximate to 1. Therefore, the correlation between the IP and *σ*_p_ is better than that between the IP and *σ*_m_, and the IP of the substituted isoflavonoids can be better predicted by *σ*_p_. IP values computed for the substituted isoflavonoids in the gas, benzene, and water phases are plotted against *σ*_p_ in Figure 4. Equations obtained from the linear regression are also placed in Figure 4. They can be used to predict the IP and antioxidative activity of the substituted isoflavonoids by the *σ*_p_. This can be useful in the selection of suitable candidates for the synthesis of novel isoflavonoid derivatives with enhanced antioxidative properties.

In Figure 2, it can also be seen that the correlation between IP and *R* is much better than that between IP and *F*. Therefore, the electronic effect of the substituents on the IP is mainly governed by the resonance effect.

### 2.3. Proton Affinity

In a SPLET mechanism, the first step is proton transfer. This step is significant for a SPLET mechanism and is characterized by PA. The PA influences the effectiveness of the proton transfer reaction. The lower the PA, the more important the role of the corresponding O−H group and the stronger the antioxidative activity. The computed gas, benzene, and water phase PAs of 4′−OH, 5−OH, and 7−OH for the basic structure and substituted isoflavonoids are listed in Table 4. As can be seen in Table 4, the lowest PAs are at 7−OH for the investigated compounds, irrespective of the studied media. Therefore, the 7−OH is the group most likely to undergo the first step of SPLET mechanism. In addition, the strongest antioxidative group of isoflavonoid is not altered by the substituents placed at C2, regardless of the studied media.

The Pearson correlation coefficients between the *σ*_m_/*σ*_p_ and the PAs are drawn as histograms in Figure 2. In Figure 2, all of the *P*(_PA,*σ*_) are negative and the absolute value of *P*(_PA,*σ*_) is larger than 0.8. Hence, the PAs are negatively related to and highly relevant to the Hammett sigma constants. The negative value of the coefficient also characterizes how the electron-donating groups in the C2 position raise the PA, while electron-withdrawing groups have an opposite effect. The obtained results can be interpreted to say that electron-withdrawing groups in the C2 position can stabilize the anion and destabilize the neutral molecule. Hence, they decrease the O−H PA. However, electron-donating groups in the C2 position have an opposite effect, and their presence would lead to an increase in the O−H PA. Therefore, the electron-withdrawing groups placed at the C2 position would strengthen the antioxidative activity of isoflavonoid, while the electron-donating groups have an opposite effect.

**Figure 5:**
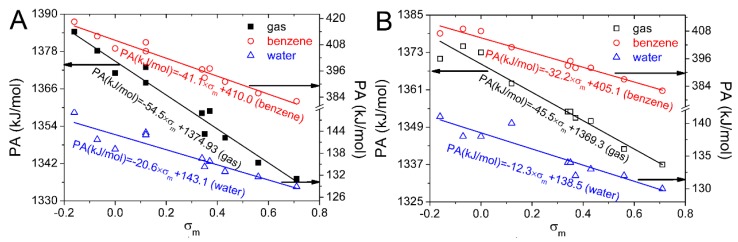
Dependence of PA on Hammett sigma constants for 4′,5,7-trihydroxyisoflavone (**A**) and 4′,5,7-trihydroxyisoflavanone (**B**) in gas, benzene, and water phases. The black arrows indicate the corresponding vertical coordinate of the relevant line.

For 4′−OH, the absolute value of *P*(_PA, *σ*p_) is larger than that of *P*(_PA, *σ*m_) and exceeds 0.9. Meanwhile, for 5−OH and 7−OH, the absolute value of *P*(_PA, *σ*m_) is larger than that of *P*(_PA, *σ*p_) and more than 0.9. Therefore, the PA(4′−OH) better correlates with *σ*_p_ and the PA(4′−OH) in the substituted isoflavonoid can be better predicted by *σ*_p_. At the same time, the opposite result occurs for 5−OH and 7−OH and their PA values in substituted isoflavonoid can be better predicted by *σ*_m_. PA values of 7−OH computed for the substituted isoflavonoids in the gas, benzene, and water phases are plotted against Hammett sigma constants in Figure 5. Equations obtained from the linear regression are also placed in Figure 5. They can be used to predict the PAs of 7−OH by the Hammett sigma constants. As the 7−OH is the strongest antioxidative hydroxyl group, the antioxidative activity of the substituted isoflavonoids can also be predicted by the equations. This can be useful in the selection of suitable candidates for the synthesis of novel isoflavonoid derivatives with enhanced antioxidative properties.

The Pearson correlation between the *F*/*R* and the PAs are also done and corresponding correlation coefficients are drawn as histograms in Figure 2. In Figure 2, it can be observed that the correlation between PA(4′−OH) and *R* is much better than that between PA(4′−OH) and *F*. Meanwhile, contrary results occur for the PA(5−OH) and PA(7−OH). Therefore, the electronic effect of substituent at the C2 position on the PA(4′−OH) of isoflavonoid is mainly governed by the resonance effect, while that on the PA(5−OH) and PA(7−OH) of isoflavonoid is mainly controlled by the field/inductive effect.

## 3. Discussion

By analyzing the data in Table 2, Table 3 and Table 4, it can be deduced that the BDEs of 4′−OH (the strongest antioxidative hydroxyl group following the HAT mechanism) are significantly smaller than the IPs and the PAs of 7−OH (the strongest antioxidative hydroxyl group following the SPLET mechanism) for the studied compounds in the gas and benzene phases. Therefore, in the gas and benzene phases, the HAT represents the thermodynamically favored process for the isoflavonoids and their derivatives when undergoing the free radical scavenging progress. The high solvation enthalpies of ionic particles induce a dramatic decrease of the PAs. In water phase, the PAs of 7−OH are significantly smaller than the lowest BDEs of 4′−OH and IPs for the studied isoflavonoids and their derivatives. Thus, SPLET is the preferred mechanism for the isoflavonoids and their derivatives when undergoing the free radical scavenging progress in water phase.

In HAT and SPLET mechanisms, the antioxidative activity of phenolic compounds is characterized by the lowest BDE and lowest PA, respectively. In the gas and benzene phases, the electron-donating substituents placed at the C2 position would enhance the antioxidative activity of isoflavonoids via a weakening of the BDE of 4′−OH, while the electron-withdrawing groups have an opposite effect. In water phase, the electron-donating substituents placed at the C2 position would weaken the antioxidative activity of isoflavonoids via an enlarging of the PA of 7−OH, while the electron-withdrawing groups have an opposite effect. Among the studied compounds, the NH_2_-substituted derivative is the strongest antioxidant in the gas and benzene phases, while the NO_2_-substituted derivative is the strongest one in water phase.

## 4. Materials and Methods 

### 4.1. Computational Details

The details of HAT (Equation (1)), SET-PT (Equations (2) and (3)), and SPLET (Equations (4) and (5)) mechanisms [7,8,9] are as follows:

R• + ArOH → RH + ArO•(1)

R• + ArOH → R^−^ + ArOH^+^• (2)

R^−^ + ArOH^+^• → RH + ArO• (3)

ArOH → ArO^−^ + H^+^ (4)

ArO^−^ + R• → ArO• + R^−^ (5)

In the Equations (1)–(5), R•, ArOH, ArO•, ArOH^+^• and ArO^−^ represent the free radical, the flavonoid, the flavonoid radical, the flavonoid cation radical, and the flavonoid anion respectively. The reaction enthalpies related to these mechanisms are as follows:

Equation (1): BDE (bond dissociation enthalpy). The calculated equation is:

BDE = *H*(ArO•) + *H*(H•) − *H*(ArOH) (6)

Equation (2): IP (ionization potential). The calculated equation is:

IP = *H*(ArOH^+^•) + *H*(e^−^) − *H*(ArOH) (7)

Equation (3): PDE (proton dissociation enthalpy). The calculated equation is:

PDE = *H*(ArO•) + *H*(H^+^) − *H*(ArOH^+^•) (8)

Equation (4): PA (proton affinity). The calculated equation is:

PA = *H*(ArO^−^) + *H*(H^+^) − *H*(ArOH) (9)

Equation (5): ETE (electron transfer enthalpy). The calculated equation is:

ETE = *H*(ArO•) + *H*(e^−^) − *H*(ArO^−^) (10)

In Equations (6)–(10), the gas and solvent phases enthalpies of H^+^, e^−^, and H• were collected from the literature [25,26,27].

The geometries of each compound and respective radical structure were optimized using the DFT method with M062X functional and the 6-311 + G^∗∗^ basis set in the gas phase and solvent phase. Single point calculations were performed using the M062X/6-311 + G^∗∗^ method. The optimized structures were confirmed to be real minima by no imaginary frequency. The solvation effects were computed by using the solvation model based on density (SMD) solvation model. All calculations were performed using Gaussian 09 program package [28].

### 4.2. Statistics Analysis

The Pearson correlation is based on the covariance matrix of the data. It is an effective method for studying a broad class of relationships among variables [29]. The Pearson correlation coefficient is the parameter evaluating the strength of relationship between two vectors. The calculated equation for the Pearson correlation coefficient (*P*_x,y_) between two vectors *x* and *y* is:

(11) $$P_{x,y}=\frac{\sum xy-\frac{\sum x\sum y}{N}}{\sqrt{(\sum x^{2}-\frac{\left(\sum x^{2}\right)}{N})\left(\sum y^{2}-\frac{\left(\sum y^{2}\right)}{N}\right)}}$$

where *N* refers to the size of the signature array. It is 11 in this work. The Pearson correlation coefficient is symmetrical: *P*_x,y_ = *P*_y,x_.

The *P*_x,y_ is among 1 to −1. The correlation between x and y is distinguished by the following criterions [29]:

(1) 0.00 < |*P*_x,y_| < 0.19: very low correlation;

(2) 0.20 < |*P*_x,y_| < 0.39: low correlation;

(3) 0.40 < |*P*_x,y_| < 0.69: moderate correlation;

(4) 0.70 < |*P*_x,y_| < 0.89: highly correlation;

(5) 0.90 < |*P*_x,y_| < 1.00: very highly correlation.

The *P*_x,y_ equal to 1 or −1 corresponds to data points lying exactly on a line, or to a bivariate distribution entirely supported on a line.

## 5. Conclusions

Understanding the role of substituents is of great importance for the preparation of novel phenolic compounds with enhanced antioxidative properties. In this paper, the effects of various substituents on reaction enthalpies related to HAT, SET-PT, and SPLET mechanisms of isoflavonoid derivations were investigated by the DFT method. The strongest antioxidative group of isoflavonoid is not altered by the substituents placed at the C2 position. Excellent correlations were found between the BDE/IP/PA and Hammett sigma constants. Equations obtained from the linear regression can be useful in the selection of suitable candidates for the synthesis of novel isoflavonoid derivatives with enhanced antioxidative properties. It is also found that:

(1) The substituent effects on BDE and IP are different than those on PA. The electron-withdrawing groups enhance BDE and IP, while the electron-donating groups reduce the BDE and IP. The effect of the substituents on PA is contrary to this.

(2) The electronic effect of the substituent on the BDE of isoflavone, IP, and PA of 4′−OH is mainly governed by the resonance effect, while that on the BDE of isoflavanone and PA of 5−OH and 7−OH is mainly controlled by the field/inductive effect.

(3) In gas and benzene phases, the free radical scavenging progress of the investigated compounds will most likely undergo the HAT mechanism. In water phase, SPLET is the most favorable mechanism.

(4) In the gas and benzene phases, the electron-donating substituents placed at the C2 position would enhance the antioxidative activity of isoflavonoids via a weakening of the BDE of 4′−OH. In water phase, they will reduce antioxidative activity by strengthening the PA of 7−OH. Contrary results occur for the electron-withdrawing group. Moreover, the NH_2_–substituted derivative is the strongest antioxidant in the gas and benzene phases, while the NO_2_–substituted derivative is the strongest antioxidant in water phase.

## Figures and Tables

**Table 1:** The Hammett sigma constants (*σ*_m_ and *σ*_p_) ^a^, the field/inductive parameter (*F*) ^a^ and the resonance parameter (*R*) ^a^ of the substituents.

	*σ*_m_	*σ*_p_	*F*	*R*
H	0.00	0.00	0.00	0.00
NH_2_	−0.16	−0.66	0.08	−0.74
OMe	0.12	−0.27	0.29	−0.56
Me	−0.07	−0.17	0.01	−0.18
OH	0.12	−0.37	0.33	−0.70
F	0.34	0.06	0.45	−0.39
Cl	0.37	0.23	0.42	−0.19
CHO	0.35	0.42	0.33	0.09
CF_3_	0.43	0.54	0.38	0.16
CN	0.56	0.66	0.51	0.15
NO_2_	0.71	0.78	0.65	0.13

^a^ Data from work by Hansch, Leo, and Taft [13].

**Table 2:** O–H bond dissociation enthalpy (BDE) in kJ/mol obtained by the M062X/6-311 + G** method ^a^.

	Gas						Benzene						Water					
	4′−OH		5−OH		7−OH		4′−OH		5−OH		7−OH		4′−OH		5−OH		7−OH	
	1	2	1	2	1	2	1	2	1	2	1	2	1	2	1	2	1	2
H	**362**	**366**	433	428	449	391	**364**	**367**	428	424	413	395	**357**	**361**	393	392	410	393
NH_2_	**359**	**362**	431	426	447	391	**360**	**364**	426	423	396	395	**352**	**359**	392	391	394	393
OMe	**357**	**363**	431	428	449	391	**359**	**366**	427	425	406	395	**353**	**361**	392	393	398	394
Me	**363**	**364**	432	427	448	391	**365**	**366**	427	423	408	395	**359**	**360**	393	391	400	393
OH	**359**	**364**	433	429	449	392	**361**	**366**	427	425	406	396	**354**	**362**	393	393	401	395
F	**364**	**367**	434	429	452	394	**367**	**369**	429	425	417	398	**361**	**364**	394	394	406	396
Cl	**365**	**367**	435	429	453	394	**368**	**369**	431	426	428	399	**363**	**364**	395	395	409	396
CHO	**364**	**366**	436	429	452	393	**366**	**368**	430	426	426	397	**361**	**362**	396	394	410	395
CF_3_	**368**	**368**	437	430	454	395	**371**	**370**	432	426	440	399	**366**	**364**	397	394	412	396
CN	**370**	**371**	439	430	454	396	**373**	**373**	433	426	446	400	**368**	**366**	397	395	415	397
NO_2_	**369**	**372**	440	430	455	397	**372**	**374**	434	426	458	401	**367**	**367**	398	395	418	398

^a^ The data in bold and underline represents the lowest BDEs.

**Table 3:** Ionization potential (IP) in kJ/mol obtained by the M062X/6-311 + G** method.

	Gas		Benzene		Water	
	1	2	1	2	1	2
H	758	793	649	680	563	587
NH_2_	722	784	614	668	533	580
OMe	718	787	618	673	544	585
Me	754	787	647	676	566	586
OH	734	789	632	675	550	585
F	764	807	657	687	571	589
Cl	769	805	664	686	581	588
CHO	769	805	662	689	579	593
CF_3_	788	806	679	687	591	591
CN	792	817	682	695	591	593
NO_2_	795	816	686	695	593	598

**Table 4:** Proton affinity (PA) in kJ/mol obtained by the M062X/6-311 + G** method ^a^.

	Gas						Benzene						Water					
	4′−OH		5−OH		7−OH		4′−OH		5−OH		7−OH		4′−OH		5−OH		7−OH	
	1	2	1	2	1	2	1	2	1	2	1	2	1	2	1	2	1	2
H	1415	1423	1445	1445	**1371**	**1373**	445	450	470	470	**406**	**408**	166	165	163	162	**139**	**138**
NH_2_	1422	1433	1461	1443	**1384**	**1371**	452	459	486	469	**419**	**407**	169	168	177	162	**149**	**141**
OMe	1416	1431	1451	1438	**1373**	**1366**	450	455	477	465	**409**	**403**	168	166	171	160	**144**	**138**
Me	1419	1422	1452	1447	**1378**	**1375**	449	451	477	471	**412**	**409**	167	166	167	162	**142**	**138**
OH	1434	1431	1445	1435	**1368**	**1363**	456	455	472	464	**405**	**401**	168	165	169	161	**143**	**140**
F	1417	1421	1430	1422	**1358**	**1354**	445	448	458	452	**397**	**393**	166	164	159	155	**137**	**134**
Cl	1415	1420	1429	1420	**1359**	**1352**	445	449	457	450	**397**	**392**	167	164	158	154	**136**	**132**
CHO	1390	1404	1424	1424	**1352**	**1354**	433	439	455	455	**393**	**395**	165	163	157	157	**134**	**134**
CF_3_	1398	1417	1419	1419	**1350**	**1351**	435	447	450	450	**391**	**392**	166	163	154	154	**133**	**133**
CN	1384	1410	1411	1410	**1342**	**1342**	425	443	444	445	**386**	**387**	166	163	153	154	**132**	**132**
NO_2_	1377	1409	1404	1403	**1337**	**1337**	423	442	439	439	**382**	**382**	165	163	149	149	**129**	**130**

^a^ The data in bold and underline represents the lowest PAs.

## Abbreviations

DFT	Density Functional Theory
HAT	Hydrogen Atom Transfer
SPLET	Sequential Proton Loss Electron Transfer
SET-PT	Single Electron Transfer Followed by Proton Transfer
ArOH	Flavonoid
BDE	Bond Dissociation Enthalpy
IP	Ionization Potential
PDE	Proton Dissociation Enthalpy
PA	Proton Affinity
ETE	Electron Transfer Enthalpy
*σ*	Hammett sigma constants
*F*	field/inductive effect
*R*	resonance effect
*P*	Pearson correlation coefficient
